# Linking preclinical models to clinical realities: VEGF/VEGFR inhibitors and thrombotic microangiopathy in cancer therapy

**DOI:** 10.1002/imo2.70014

**Published:** 2025-03-28

**Authors:** Aimin Jiang, Zhanzhi Li, Ying Liu, Junyi Shen, Quan Cheng, Anqi Lin, Peng Luo, Linhui Wang

**Affiliations:** ^1^ Department of Urology, Changhai Hospital Naval Medical University (Second Military Medical University) Shanghai China; ^2^ School of Clinical Medicine Hangzhou Medical College Hangzhou China; ^3^ Department of Oncology, Zhujiang Hospital Southern Medical University Guangzhou China; ^4^ Department of Neurosurgery, Xiangya Hospital Central South University Changsha Hunan China; ^5^ National Clinical Research Center for Geriatric Disorders, Xiangya Hospital Central South University Changsha Hunan China

**Keywords:** drug‐induced thrombotic microangiopathy, VEGF inhibitors, VEGF receptor inhibitors

## Abstract

This study examines the link between vascular endothelial growth factor inhibitors (VEGFi) and VEGF receptor inhibitors (VEGFRi) used in treating malignant tumors and the incidence of thrombotic microangiopathy (TMA). Understanding TMA's clinical features and mechanisms is essential for its management due to its severe impacts. This study analyzed data from the FDA's Adverse Event Reporting System (FAERS) and WHO's global pharmacovigilance database (Vigibase) to assess the risk of TMA associated with VEGF and VEGFR inhibitors. We also examined TMA and thrombotic thrombocytopenic purpura (TTP) risks using patient biochemical data from a local hospital and explored underlying biological mechanisms through animal models and pan‐cancer analysis. Our study confirms that VEGFi and VEGFRi elevate the risk of TMA. Notably, Bevacizumab, Sunitinib, Ramucirumab, and Aflibercept significantly increase TMA risks, with Bevacizumab showing the highest risk (reporting odds ratio 4.96 [4.08–6.03] in Vigibase and 2.33 [1.84–2.94] in FAERS). Biochemical analysis from 1698 patients indicated impaired kidney function and hemolytic events, confirming that VEGFi and VEGFRi significantly increase the risk of TMA/TTP in clinical use (*p* < 0.001). Animal studies highlighted that Semaxanib causes more severe endothelial damage and thrombus formation than Bevacizumab, further validating that VEGFi typically induces TMA later than VEGFRi. Transcriptomic analysis and pan‐cancer pathway insights identified critical pathways involving reduced VEGF signaling, abnormal complement activation, and excessive platelet aggregation leading to thrombosis. The results underscore the enhanced risk of TMA posed by these inhibitors, particularly noting the timelines and mechanisms through which different inhibitors trigger TMA, and recommend regular monitoring of biochemical markers for early risk assessment and management.

## Highlights

- First comprehensive risk assessment linking vascular endothelial growth factor (VEGF) and vascular endothelial growth factor receptor (VEGFR) inhibitors (e.g., Bevacizumab, Sunitinib) to thrombotic microangiopathy (TMA) in cancer therapy using the FDA Adverse Event Reporting System (FAERS) and the WHO Global Database for Adverse Drug Reactions (Vigibase) databases.
- Mechanistic validation via animal models: VEGFR inhibitors induce acute endothelial damage via complement activation, while VEGF inhibitors trigger delayed TMA through VEGF signaling suppression.
- Clinical relevance was confirmed by biochemical data from 1698 patients, showing posttreatment platelet decline (*p* < 0.001) and hemolysis markers elevation.
- Pan‐cancer pathway analysis identifies platelet aggregation and complement activation as key drivers of TMA, informing targeted monitoring and therapeutic strategies.
- Practical recommendations: Regular monitoring of renal function and platelet counts for early TMA detection in patients receiving antiangiogenic therapies.

## Graphical abstract

This study investigates the risk of thrombotic microangiopathy (TMA) induced by vascular endothelial growth factor (VEGF) and vascular endothelial growth factor receptor (VEGFR) inhibitors in cancer therapy. Using data from the FDA Adverse Event Reporting System (FAERS), the WHO Global Database for Adverse Drug Reactions (Vigibase), and The Cancer Genome Atlas (TCGA), along with clinical analysis of 1,698 patients and animal experiments, it reveals that VEGFR inhibitors cause rapid endothelial damage through complement activation, while VEGF inhibitors trigger delayed TMA via suppressed VEGF signaling. The analysis of TCGA pan‐cancer transcriptomic data identified critical pathways involved, such as platelet activation and complement cascade, contributing to TMA development. Animal model studies further confirmed these findings, with hematoxylin and eosin (HE) staining of kidney tissues showing significant endothelial damage, thrombus formation, and mesangial cell proliferation, reinforcing the biological mechanisms. This comprehensive approach underscores the need for regular monitoring of renal function and platelet counts in patients receiving VEGF/VEGFR inhibitors to mitigate TMA risks.

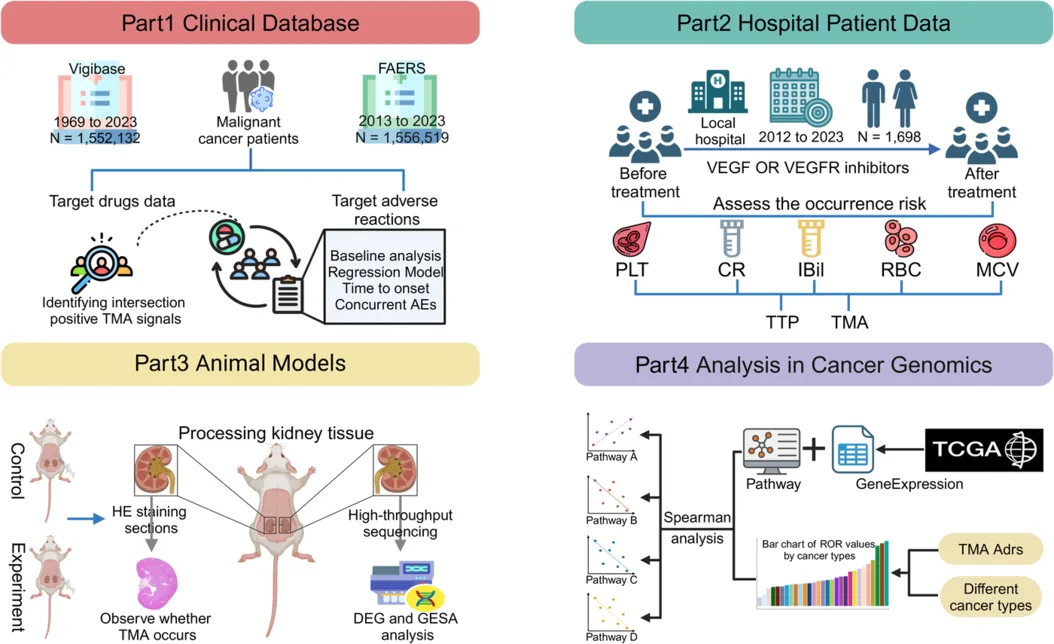

## 1 INTRODUCTION

Thrombotic microangiopathy (TMA) comprises a heterogeneous group of disorders characterized by microvascular thrombosis, mechanical hemolysis of red blood cells, and platelet consumption, predominantly affecting the kidneys [1]. These conditions typically encompass renal‐limited TMA, thrombotic thrombocytopenic purpura (TTP), microangiopathic hemolytic anemia (MAHA), hemolytic uremic syndrome (HUS), and atypical HUS (aHUS) [2]. The pathophysiological mechanisms of TMA primarily involve endothelial cell injury and activation, leading to platelet aggregation and microthrombi formation at sites of vascular damage, with concurrent mechanical hemolysis of red blood cells. Currently, TMA is classified into various types, including genetic, acquired, and pregnancy‐associated forms [3]. The impact of TMA is profound, with potential outcomes ranging from acute to chronic renal damage, neurological symptoms, and systemic manifestations, including fatigue and jaundice, which severely threaten patient survival and quality of life.

Over the past decade, significant advancements in the development of drugs targeting vascular endothelial growth factor (VEGF) have positioned these agents as a crucial strategy in cancer treatment by primarily inhibiting tumor angiogenesis [4]. For instance, bevacizumab, which prevents VEGF from binding to its receptors, and sunitinib, which inhibits VEGFR signaling, have demonstrated significant efficacy in treating various solid tumors [5]. In treating metastatic renal cell carcinoma, combining bevacizumab with atezolizumab significantly extended progression‐free survival (PFS) from 7.7 to 11.2 months (hazard ratio (HR) = 0.74, 95% CI [0.57–0.96]), and reduced the mortality risk for overall survival (OS) by 7% (HR = 0.93, 95% CI [0.76–1.14]) compared to the sunitinib group [6]. Similarly, sunitinib significantly prolonged the PFS and OS in the treatment of gastrointestinal stromal tumors, with median values rising from 6.4 weeks in the placebo group to 27.3 weeks (HR = 0.33, 95% CI [0.23–0.47]), and a significant 51% reduction in the risk of death (HR = 0.49, 95% CI [0.29–0.83]) [7]. However, despite the evident clinical benefits of these drugs, they are associated with a range of potential side effects, particularly renal adverse effects such as proteinuria, hypertension, and kidney damage, which limit their use [8]. Drug‐induced TMA (DITMA) constitutes 10%–13% of all TMA cases and is particularly common among drugs used in cancer, including VEGF and VEGFR inhibitors, representing a recognized severe complication in oncology [9, 10]. Although relatively rare, DITMA can severely impact cancer patients, potentially proving fatal [11].

The earliest case reports of TMA induced by the use of VEGF and VEGFR inhibitors appeared in 2007 [12, 13]. A study published in *Lancet Oncology* reported a case of TMA in a patient with metastatic renal cell carcinoma treated with bevacizumab [12]. Another case described in *Annals of Oncology* documented the first instance of TMA in a patient using sunitinib [13]. Subsequently, more studies focused on descriptive analyses and small‐scale observations [14], with further exploration into the clinical manifestations and potential mechanisms of drug‐induced TMA [15]. However, current research primarily focuses on case reports of TMA caused by bevacizumab and sunitinib. Research remains significantly lacking regarding the risk of TMA associated with other VEGF and VEGFR inhibitors. Moreover, although existing literature has documented cases of TMA caused by these drugs [16], current studies have not systematically compared the differences between VEGF and VEGFR inhibitors in inducing TMA, nor have they comprehensively assessed clinical factors in patients, from the duration of drug use to the onset of TMA, which limits effective guidance in clinically preventing TMA.

Therefore, this study aims to address existing research gaps by identifying VEGF and VEGFR inhibitors that pose a high risk of TMA, utilizing real‐world adverse reactions (AEs) data from the FDA's Adverse Event Reporting System (FAERS) and WHO's global pharmacovigilance database (Vigibase). Additionally, it assesses clinical factors associated with the occurrence of TMA. Moreover, biochemical data from local hospitals will be utilized to evaluate the potential risks of TMA and TTP following treatment with VEGF and VEGFR inhibitors in real clinical settings. Finally, by employing a mouse model, this study further investigates the pathways through which VEGF and VEGFR inhibitors induce TMA [17], offering targeted guidance for preventing TMA in clinical practice.

## 2 RESULTS

### Baseline analysis

**FIGURE 1:**
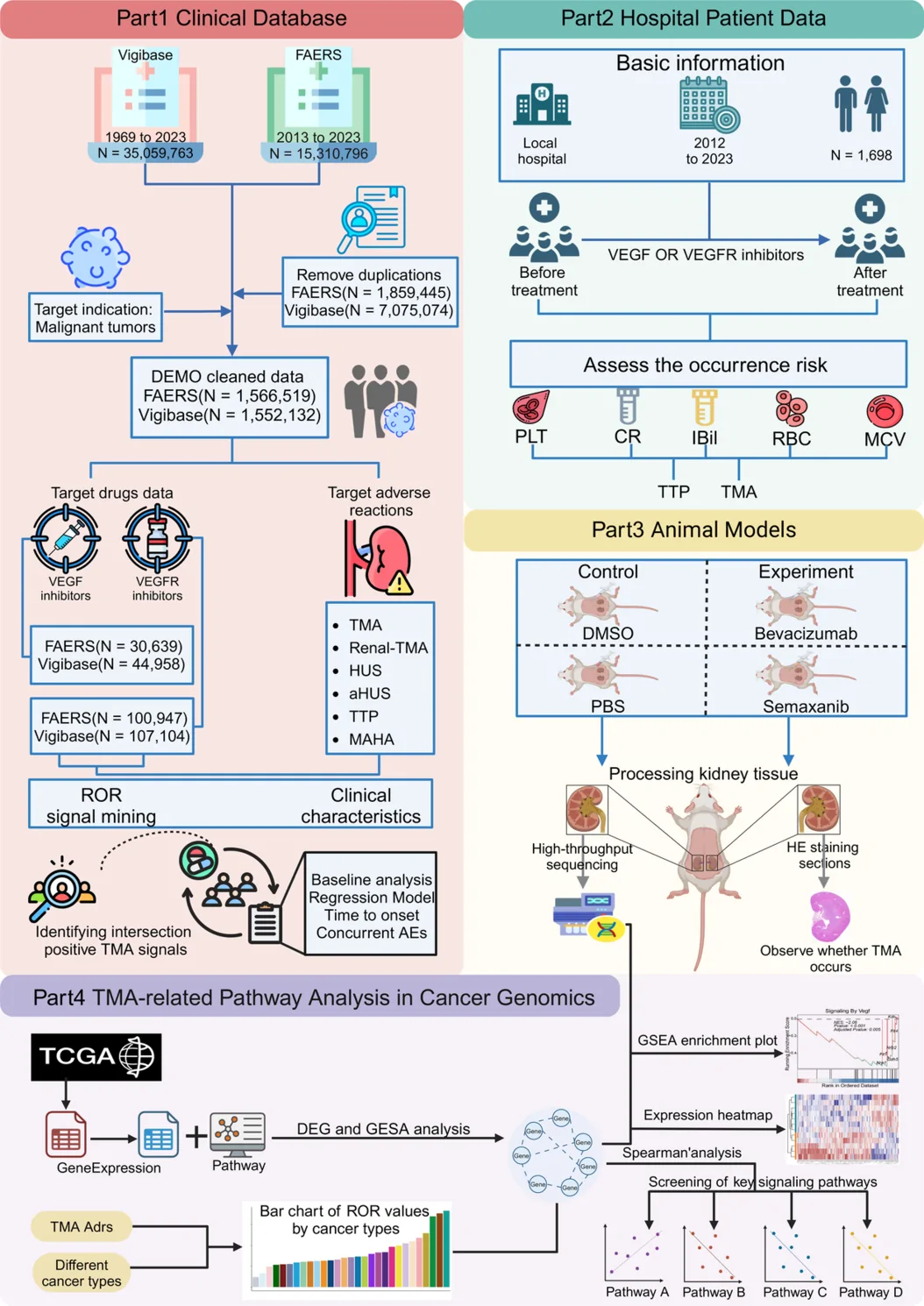
Multidimensional research framework integrating pharmacovigilance, clinical biomarkers, animal models, and transcriptomic analysis. The flowchart illustrates the multidimensional research framework of this paper, from pharmacovigilance analysis using the FDA's Adverse Event Reporting System (FAERS) and Vigibase databases, to evaluation of thrombotic microangiopathy (TMA) and thrombotic thrombocytopenic purpura (TTP) using biochemical markers in patients from a local hospital, validation through hematoxylin and eosin (HE) staining in animal experiments, and bioinformatics analysis using the cancer genome atlas (TCGA) and transcriptomic data.

Figure 1 presents the research framework, outlining the study's methodology from data collection to analysis. Patient information was collected from the FAERS and Vigibase databases for individuals who used VEGF or VEGFR inhibitors and reported incidents of TMA (Table S2). In the FAERS database, females constituted 26.7%, males 27.2%, and unknown gender 46.1%; in Vigibase, the corresponding figures were 52.5% for females, 39.8% for males, and 7.7% for unknown gender. Clinical outcomes revealed that nonfatal outcomes comprised 84.1% of cases in FAERS and 58.5% in Vigibase. Geographically, the Americas accounted for 58.1% in Vigibase, whereas FAERS showed a more balanced distribution, with Europe at 36.6% and the Americas at 29.3%. In both databases, the primary organs affected by cancer among patients were the kidneys, colon, and ovaries.

### Clinical database analysis

**FIGURE 2:**
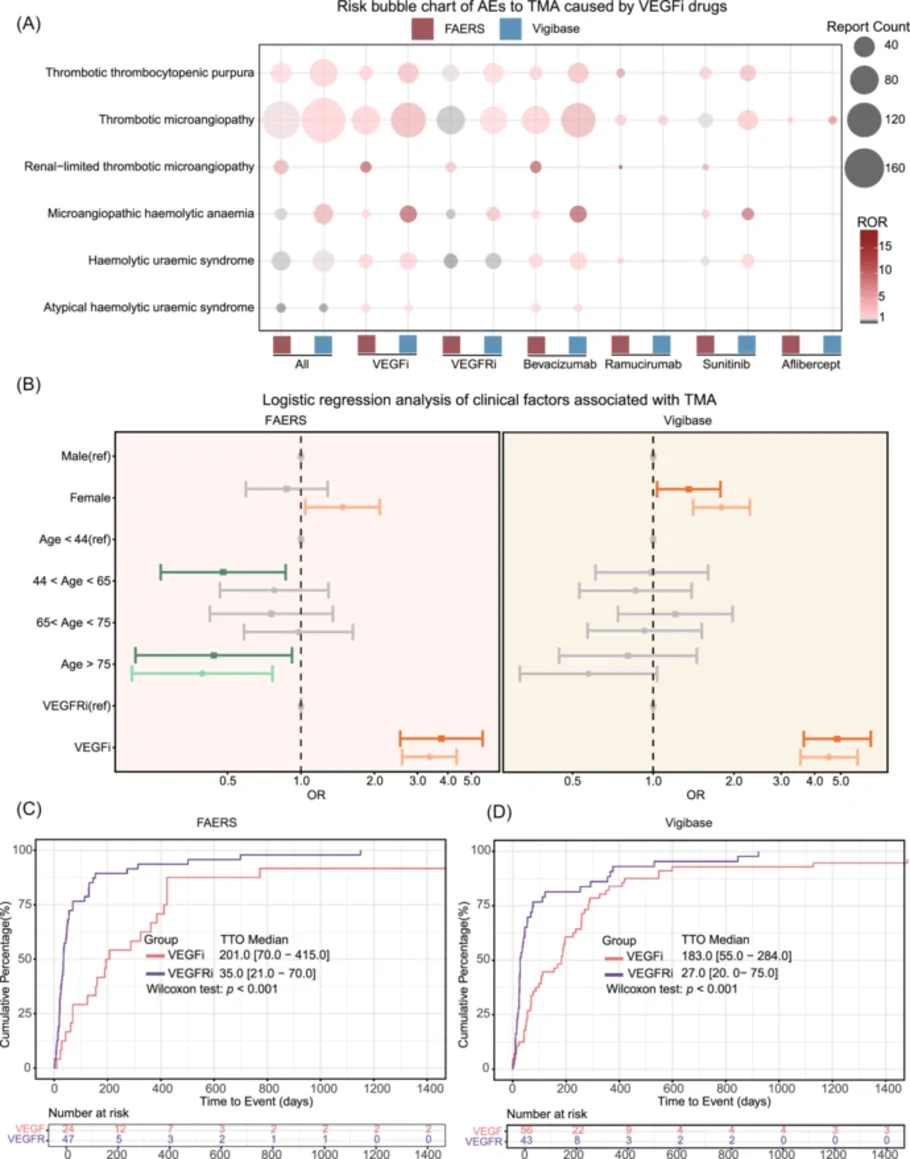
Analysis of TMA‐related reporting odds ratio (ROR) and time to onset (TTO) for vascular endothelial growth factor inhibitors (VEGFis) and vascular endothelial growth factor receptor inhibitors (VEGFRis). (A) Risk bubble diagram of TMA‐related adverse events caused by VEGF and VEGFR inhibitors, where the size of the bubbles represents the number of reports and the color indicates the ROR value. (B) Forest plot showing clinical factors related to TMA occurrence in FAERS and Vigibase, with point estimates and 95% confidence intervals represented in different colors: gray for no effect, orange for increased risk (lower limit of the confidence interval greater than 1), and green for reduced risk (lower limit of the confidence interval less than 1). Univariate logistic regression results are indicated by light‐colored circle centers, and multivariate logistic regression results by dark‐colored squares. (C, D) Cumulative percentage diagrams display the distribution differences in the timing of TMA occurrence among patients treated with VEGF and VEGFR inhibitors in FAERS and Vigibase.

In our study, we analyzed both VEGF and VEGFR inhibitors (All) collectively. The reporting odds ratio (ROR) in FAERS was 1.07 [95%CI: 0.90–1.27], while in Vigibase, it was 2.13 [95%CI: 1.80–2.51] (Figure 2A). Breaking down these inhibitors, the ROR for VEGF inhibitors was significantly higher at 4.96 [95%CI: 4.08–6.03] in Vigibase and 2.33 [95%CI: 1.84–2.94] in FAERS. In contrast, the ROR for VEGFR inhibitors was lower, at 1.20 [95%CI: 0.93–1.54] in Vigibase and 0.69 [95%CI: 0.54–0.87] in FAERS. Specific drugs like Bevacizumab, Sunitinib, Ramucirumab, and Aflibercept showed positive RORs for TMA‐related AEs. Notably, Bevacizumab was associated with a significantly higher incidence of MAHA in Vigibase, with an ROR of 13.23 [95%CI: 8.04–21.75] (Table S4).

Our logistic regression analysis in both FAERS and Vigibase assessed the impact of gender, age, and drug type on TMA occurrence (Figure 2B). In FAERS, univariate analysis revealed that women had a higher risk of TMA compared to men (*OR* = 1.48 [95%CI: 1.04–2.10], *p* = 0.029), while multivariate analysis showed no significant difference (*OR* = 0.87 [95%CI: 0.59–1.29], *p* = 0.494). Conversely, in Vigibase, univariate analysis indicated that women had a significantly higher risk of TMA than men (*OR* = 1.80 [95%CI: 1.41–2.30], *p* < 0.001), and multivariate analysis confirmed this finding (*OR* = 1.36 [95%CI: 1.03–1.78], *p* = 0.028). Concerning age, FAERS data indicated that patients over 75 had a significantly lower risk of TMA in the univariate analysis compared to the 0–44 age reference group (*OR* = 0.39 [95%CI: 0.20–0.76], *p* = 0.006), with a similar downward trend in multivariate analysis (*OR* = 0.44 [95%CI: 0.21–0.92], *p* = 0.029). However, Vigibase data revealed no significant association between age and TMA risk. Regarding drug type, patients using VEGF inhibitors in FAERS showed a significantly increased risk of TMA compared to those using VEGFR inhibitors (*OR*_ULR_ = 3.36 [95%CI: 2.60–4.35], *p* < 0.001; *OR*_MLR_ = 3.76 [95%CI: 2.55–5.54], *p* < 0.001). Results in Vigibase also showed that the use of VEGF inhibitors was associated with a higher risk of TMA (*OR*_ULR_ = 4.52 [95%CI: 3.54–5.78], *p* < 0.001; *OR*_MLR_ = 4.85 [95%CI: 3.64–6.46], *p* < 0.001).

Figure 2C from the FAERS and Vigibase databases shows TMA onset times among patients using VEGF and VEGFR inhibitors, differentiated by gender and drug type. In FAERS, the time to onset (TTO) for TMA with VEGF inhibitors averaged 201.0 days [interquartile range (IQR) 70.0‐415.0], significantly longer than VEGFR inhibitors at 35.0 days [IQR 21.0–70.0] (*p* < 0.001). Vigibase reported similar trends, with VEGF inhibitors at 183.0 days [IQR 55.0–284.0] and VEGFR inhibitors at 27.0 days [IQR 20.0–75.0]. Gender did not significantly affect TMA onset times, with FAERS recording 25.0 days [IQR 17.0–159.0] for females and 55.0 days [IQR 29.0–152.0] for males, and Vigibase showing 75.0 days [IQR 25.0–258.0] for females and 68.0 days [IQR 41.0–258.0] for males (Figure S1).

We also reviewed concomitant AEs in 232 FAERS patients using these inhibitors (Figure S2), noting hypertension (*n* = 34), proteinuria (*n* = 32), nephrotic syndrome (*n* = 23), acute kidney injury (*n* = 21), and thrombocytopenia (*n* = 22) as the most common. Other noted AEs included impaired renal function (*n* = 12), renal failure (*n* = 11), segmental glomerulosclerosis (*n* = 9), hematuria (*n* = 6), and renal tubular necrosis (*n* = 5).

### Local patient risk assessment

**FIGURE 3:**
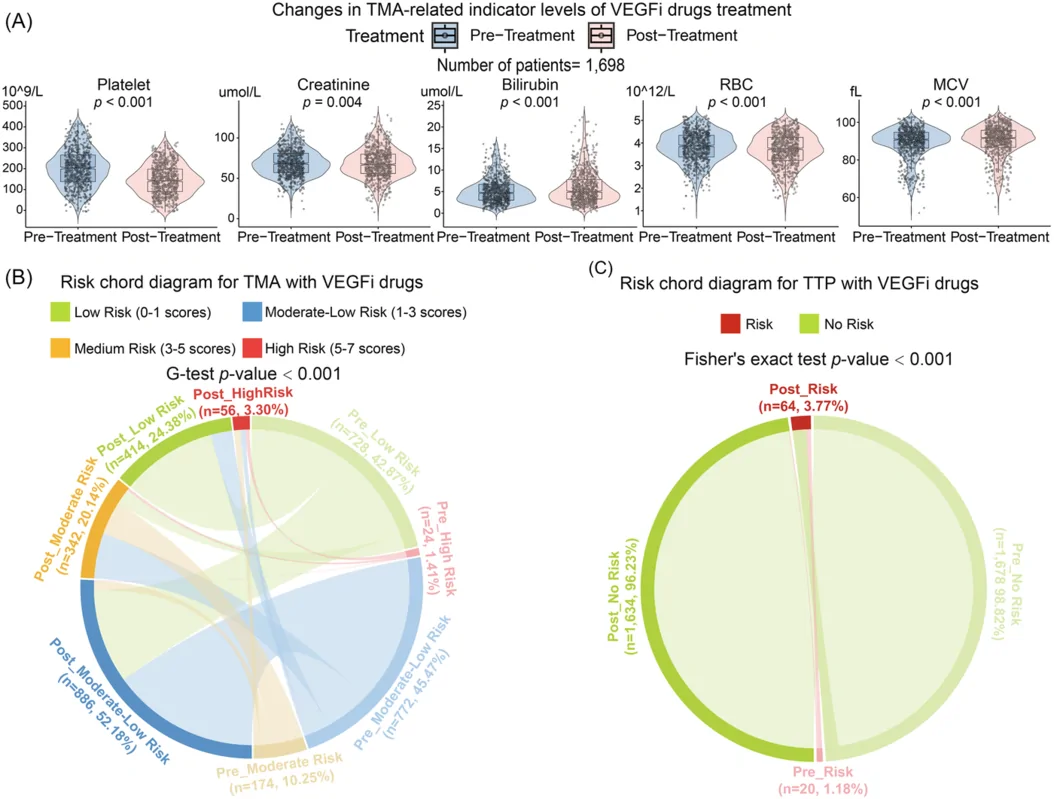
Changes and risk assessment of thrombotic microangiopathy (TMA)‐related indicators in patients before and after treatment with VEGFis and VEGFRis. (A) Violin box plots illustrate the changes in creatinine, platelet count, red blood cell (RBC) count, bilirubin, and mean corpuscular volume (MCV) before and after treatment. (B) Chord diagrams show changes in TMA risk levels in patients before and after treatment. (C) Chord diagrams show changes in TTP risk levels in patients before and after treatment. TTP, thrombotic thrombocytopenic purpura; VEGFis, vascular endothelial growth factor inhibitors; VEGFRis, VEGF receptor inhibitors.

Figure 3A shows changes in TMA‐related indicators among 1698 patients treated with VEGFi at Southern Medical University's Zhujiang Hospital. The mean platelet count dropped from 233.4 × 10^9/L to 171.1 × 10^9/L, with the median decreasing from 223.0 × 10^9/L to 159.0 × 10^9/L (*p* < 0.001). Mean creatinine levels slightly increased from 76.4 to 76.7 μmol/L, while the median decreased from 70.0 to 69.2 μmol/L (*p* = 0.004). Indirect bilirubin levels rose from 6.9 to 7.9 μmol/L, with median values increasing from 4.9 to 5.4 μmol/L (*p* < 0.001). Mean red blood cell count (RBC) count decreased from 3.9 × 10^12/L to 3.8 × 10^12/L, with the median dropping from 3.9×10^12/L to 3.8×10^12/L (*p* < 0.001). The mean corpuscular volume (MCV) rose from 90.19 to 91.3 fL, with the median increasing from 91.4 to 92.1 fL (*p* < 0.001).

Figure 3B,C illustrates changes in TMA and TTP risk before and after treatment with VEGF or VEGFR inhibitors. Initially, 772 patients (45.47%) were at medium‐low risk and 728 (42.87%) at low risk. posttreatment, medium‐low risk increased to 886 (52.18%), and low risk decreased to 414 (24.38%), with high‐risk patients rising from 24 to 56 (1.41%–3.30%). These changes were statistically significant (G‐test, *p* < 0.001). For TTP risk in Figure 3C, patients at risk increased from 20 (1.18%) to 64 (3.77%), while those not at risk decreased from 1678 to 1634 (98.82%–96.23%), confirmed by Fisher's exact test (*p* < 0.001), suggesting that VEGF or VEGFR inhibitor treatment may elevate TTP risk.

### Impact of cancer type on TMA risk and related pathway analysis

**FIGURE 4:**
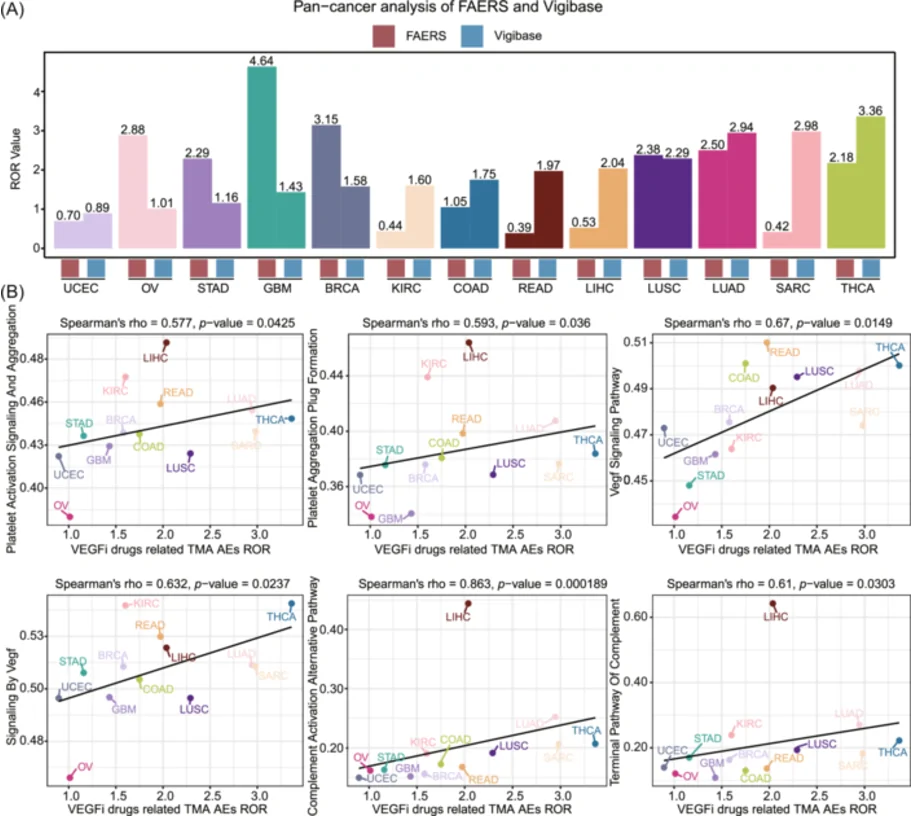
Reporting odds ratio (ROR) and key biological pathway activities of thrombotic microangiopathy (TMA) adverse events associated with VEGFis and VEGFRis across different cancer types. (A) ROR of TMA adverse reactions (AEs) associated with VEGF(R) inhibitors in 13 cancer types, with types having fewer than 3 cases excluded from analysis. (B) Analysis of the correlation between single‐sample gene set enrichment analysis (ssGSEA) enrichment scores for platelet activation and aggregation, platelet thrombus formation, the VEGF signaling pathway, the alternative complement activation pathway, and the terminal complement pathway with TMA AEs. Cancer types include Uterine Corpus Endometrial Carcinoma (UCEC), Ovarian Cancer (OV), Gastric Adenocarcinoma (STAD), Glioblastoma Multiforme (GBM), Invasive Breast Cancer (BRCA), Renal Clear Cell Carcinoma (KIRC), Colon Adenocarcinoma (COAD), Rectal Adenocarcinoma (READ), Hepatocellular Carcinoma (LIHC), Lung Squamous Cell Carcinoma (LUSC), Lung Adenocarcinoma (LUAD), Soft Tissue Sarcoma (SARC), and Thyroid Cancer (THCA). VEGFis, vascular endothelial growth factor inhibitors; VEGFRis, VEGF receptor inhibitors.

Figure 4A shows ROR values for TMA‐related AEs from VEGF or VEGFR inhibitors across 13 cancer types in the FAERS and Vigibase databases. Glioblastoma (GBM) in FAERS displayed the highest ROR at 4.64 [95%CI: 1.94–11.06], while in Vigibase, thyroid cancer (THCA) had the highest at 3.36 [95%CI: 2.04–5.55]. Both databases showed consistently high ROR values in lung squamous cell carcinoma, lung adenocarcinoma, thyroid cancer (THCA), and breast cancer (BRCA), with endometrial cancer (UCEC) showing low RORs. Figure 4B integrates Vigibase cancer type RORs with the cancer genome atlas (TCGA) pan‐cancer transcriptome data, using single‐sample gene set enrichment analysis (ssGSEA) to identify pathways linked with ROR variations. Significant correlations were found with platelet activation and aggregation (Spearman's rho = 0.577, *p* = 0.043), platelet plug formation (Spearman's rho = 0.593, *p* = 0.036), VEGF signaling (Spearman's rho = 0.67, *p* = 0.0149; Spearman's rho = 0.632, *p* = 0.024), complement activation alternative pathway (Spearman's rho = 0.863, *p* < 0.001), and complement terminal pathway (Spearman's rho = 0.61, *p* = 0.030).

### Animal experiment to verify mechanisms of TMA induction by medication

**FIGURE 5:**
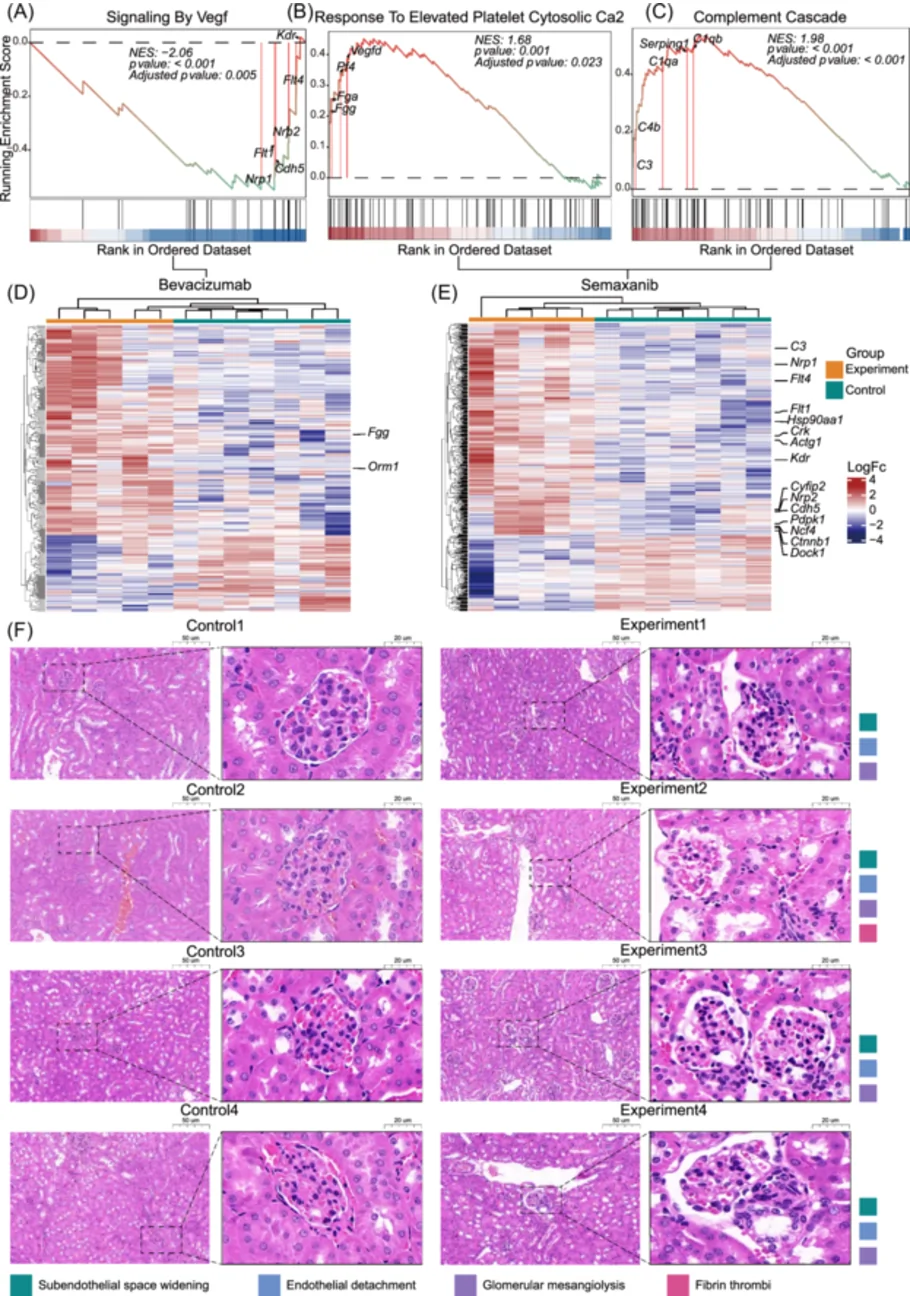
Effects of VEGFis and VEGFRis on thrombotic microangiopathy (TMA)‐related biological pathways in animal models. (A) Displays the results of gene set enrichment analysis (GSEA) for the vascular endothelial growth factor (VEGF) signaling pathway in the Bevacizumab group. (B) Displays the results of GSEA for the pathway of elevated cytosolic calcium in platelets in the Semaxanib group. (C) Displays the GSEA results for the complement cascade pathway in the Semaxanib group. (D) Heatmap showing gene expression differences between the phosphate buffered saline (PBS) control group and the Bevacizumab‐treated group. (E) Heatmap showing gene expression differences between the dimethyl sulfoxide (DMSO) control group and the Semaxanib‐treated group. (F) HE stained sections showing the renal tissues of experimental and control group mice, including typical TMA‐related pathological changes such as endothelial cell detachment, widening of the subendothelial space, formation of fibrin thrombi, and mesangiolysis.

Using GSEA, we examined the impact of VEGF and VEGFR inhibitors on biological pathways associated with TMA (Figure 5A–C). Post‐Bevacizumab treatment in the VEGF group, there was a decrease in activity in the Signaling by VEGF pathway (Enrichment Score = −0.550, Adjusted *p*‐value = 0.008). Conversely, in the VEGFR group, we noted positive enrichment in response to Elevated Platelet Cytosolic Ca2 pathway, suggesting increased platelet activation (Enrichment Score = 0.452, Adjusted *p*‐value = 0.024), and a significant activation in the Complement Cascade pathway (Enrichment Score = 0.536, Adjusted *p*‐value < 0.001) during Semaxanib treatment, indicating complement system activation. Further analysis through differential gene expression between the Bevacizumab and Semaxanib groups (Figure 5D–E) and histological examination of HE‐stained kidney sections (Figure 5F) revealed significant microstructural kidney damage in treated animals. Damage included expansion of the subendothelial area, endothelial cell detachment, mesangial cell proliferation in the glomeruli, and fibrin thrombi formation.

## 3 DISCUSSION

This study is the first to assess TMA relevance in cancer patients treated with VEGF and VEGFR inhibitors, using real‐world data from the FAERS and Vigibase databases [18]. We identified specific inhibitors linked to TMA and explored the clinical characteristics influencing these AEs [19]. Additionally, by incorporating patient data from local hospitals, we evaluated the TMA risk associated with these drugs in patients with malignant tumors [20]. Using pan‐cancer transcriptomic and animal experiment data, we investigated the biological mechanisms through which these drugs trigger TMA. Our findings highlight the need for meticulous clinical management of VEGFi inhibitors, focusing on precise risk assessments and preventive strategies, especially for high‐risk patients.

While TMA AEs are rare in the treatment of malignant tumors with VEGF and VEGFR inhibitors [21], they represent a significant risk. According to the FAERS database, from 2013 to 2023, about 0.18% of patients treated with these inhibitors reported TMA events; Vigibase data from 1969 to 2023 showed a similar incidence of 0.19%. In detailed drug assessments, six VEGF inhibitors and 16 VEGFR inhibitors were analyzed. Notably, Bevacizumab, Sunitinib, Ramucirumab, and Aflibercept were significantly linked to TMA‐related AEs, with Bevacizumab and Sunitinib posing higher risks. Subtypes of TMA, such as renal‐limited TMA, TTP, MAHA, and HUS, warrant heightened clinical vigilance. TTP typically features severe thrombocytopenia and neurological symptoms [22], MAHA involves fragmented red cells and hemolytic anemia [23], and HUS is characterized by renal dysfunction alongside hemolytic anemia and thrombocytopenia.

Further analysis showed that VEGF inhibitors carry a higher risk of inducing TMA than VEGFR inhibitors, with a delayed onset likely due to their prolonged effects on vascular physiology. Bevacizumab, in particular, has been linked to the highest incidence of TMA [24], typically appearing 6 months or more after treatment, while TMA from Sunitinib usually appears after 1 month [25]. Additionally, no significant correlations were found between TMA risk and demographic factors such as gender and age, suggesting that TMA is more dependent on the specific pharmacological actions of the drugs and individual physiological responses rather than general patient characteristics [26].

In our study, we analyzed 232 FAERS database patients who used VEGF or VEGFR inhibitors and reported TMA, uncovering severe complications such as hypertension, proteinuria, nephrotic syndrome, acute renal injury, and thrombocytopenia [27]. These AEs highlight the risks these drugs pose to vascular and renal health. Thrombocytopenia, in particular, may reflect the indirect effects of these inhibitors on vascular structures, leading to microvascular damage and TMA onset [28]. Furthermore, renal complications like impaired kidney function, renal failure, and renal tubular necrosis demonstrate the potential severe renal impacts of these treatments. These findings emphasize the need for diligent monitoring of renal function and platelet levels, along with timely management of TMA and its complications [29].

In our local patient data analysis, we confirmed that VEGF and VEGFR inhibitors significantly elevate the risk of TMA/TTP, as evidenced by substantial changes in key biochemical indicators such as decreased platelet count [18], increased creatinine levels, elevated indirect bilirubin, reduced RBC count, and lowered MCV. These indicators collectively suggest impaired renal function, hemolytic events, and microvascular damage [30]. To further understand TMA's pathological mechanisms, we recommend renal biopsies for patients suspected of having TMA to directly observe changes like thrombosis within glomerular microvessels [31]. Our study also links the activation of biological pathways across various cancer types with the ROR of TMA from VEGF and VEGFR inhibitors. We found that inhibiting the VEGF signaling pathway affects the survival and function of vascular endothelial cells, potentially compromising vascular integrity and triggering TMA. Additionally, the complement system's activation, especially through its alternative and terminal pathways [32], exacerbates local inflammation and thrombosis without antibody involvement, further damaging vascular endothelial cells [33]. Excessive platelet activation and aggregation, exacerbated by vascular injury or inflammatory signaling, also contribute to thrombus formation. Transcriptomic data from animal experiments support these findings, showing pathway activations via GSEA [34].

In the Bevacizumab group, reduced VEGF signaling pathway activity is shown by decreased expression of key angiogenesis genes like *Nrp1*, *Nrp2*, *Kdr*, *Flt1*, and *Flt4*. Conversely, the Semaxanib group displays increased platelet activation through the Response to Elevated Platelet Cytosolic Ca2 pathway, with heightened levels of platelet factor 4 (*Pf4*) and fibrinogens (*Fgg* and *Fga*), emphasizing the role of platelet activation in coagulation [35]. Furthermore, significant activation of the Complement Cascade pathway, indicated by increased expression of *C3*, *C4b*, and *Serping1*, underscores its importance in this context. Histological findings from hematoxylin and eosin (HE)‐stained sections of animal models, including widened glomerular endothelial cells, microvascular damage, and thrombus formation in mice treated with Semaxanib, support these pathway activations [36]. These pathological changes, consistent with biomarker patterns in transcriptomic data, validate the mechanisms by which VEGFR inhibitors can induce TMA more rapidly than VEGF inhibitors. Currently, treatment of TMA primarily involves plasma exchange [37]; however, our findings highlight additional therapeutic targets within the key pathways that have been identified. The complement activation pathway is particularly promising, as aberrant activation is a key factor in the development of TMA. The anti‐C5 antibody eculizumab has been shown to be useful in treating bevacizumab‐mediated TMA [38]. In addition, targeting the platelet aggregation pathway with antiplatelet drugs may help prevent thrombosis by reducing excessive platelet activation and aggregation, but further development and validation are needed.

Although our animal models demonstrated that Semaxanib induces more severe endothelial damage and thrombus formation compared to Bevacizumab, this difference is not as evident in clinical data. In our clinical analysis, VEGFR inhibitors, including Semaxanib, were associated with a significantly shorter TTO of TMA (35 days [IQR 21–70]) compared to VEGF inhibitors like Bevacizumab (201 days [IQR 70–415], *p* < 0.001). However, clinical reports of TMA related to Semaxanib are fewer and primarily categorized under VEGFR inhibitors, whereas Bevacizumab, a VEGF inhibitor, has a higher number of reported TMA cases. This discrepancy may be due to the more frequent use and broader application of VEGF inhibitors in clinical practice, as well as dosage differences and individual patient factors. Additionally, the stricter dose control in animal experiments likely led to more pronounced drug effects, resulting in more severe TMA manifestations in the preclinical models.

In this study, we used real‐world data from the FAERS and Vigibase databases to examine the link between VEGF and VEGFR inhibitors used in malignant tumors and the occurrence of TMA. We expanded our investigation by integrating pan‐cancer transcriptomic and animal experimental data to explore the biological mechanisms these drugs use to induce TMA [14]. Despite providing significant insights, our study has limitations, including potential reporting biases in the FAERS and Vigibase systems and inherent limitations of the ROR algorithm, influenced by geographical, racial, and market factors [39]. To mitigate these limitations, caution is warranted when interpreting ROR results, and future studies should incorporate additional data sources, such as electronic health records, to validate the findings. We acknowledge that our experimental findings were constrained by the utilization of healthy C57BL/6J mice rather than tumor‐bearing models, potentially limiting the generalizability of our results. The lack of tumor‐bearing models precluded comprehensive replication of the pathophysiological microenvironment characteristic of cancer patients undergoing VEGF/VEGFR inhibitor therapy. Subsequent investigations should implement specific tumor models, particularly those representing renal and colorectal malignancies, to elucidate the mechanisms underlying drug‐induced TMA in cancer‐specific contexts. Moreover, examining diverse age cohorts and multiple cancer subtypes would yield comprehensive insights into the correlation between tumor burden, cancer classification, and TMA development during therapeutic intervention. These methodological refinements would facilitate a more clinically relevant understanding of the mechanistic relationship between VEGF/VEGFR inhibition and TMA manifestation in the therapeutic oncological setting. Future research should involve large‐scale, prospective studies and include diverse animal models encompassing different sexes, age groups, and cancer types to more accurately assess the safety and efficacy of these inhibitors and better understand the mechanisms underlying TMA development [40].

## 4 CONCLUSION

This study represents the first comprehensive analysis of the association between VEGF and VEGFR inhibitors and the occurrence of TMA, utilizing real‐world data from FAERS and Vigibase, and integrating local patient data with biochemical and transcriptomic analyses of animal models. The results highlight the increased risk of TMA associated with these inhibitors, particularly noting the timelines and mechanisms through which different inhibitors trigger TMA. In clinical practice, it is recommended that patients receiving VEGF and VEGFR inhibitors undergo regular monitoring [37], including key biochemical markers such as platelet count, creatinine, and indirect bilirubin, for early risk assessment and management. If monitoring results show abnormalities or if patients exhibit symptoms of TMA, the initial step should be to suspend drug therapy [41]. Typically, after discontinuation of the medication, patients' symptoms may improve [42]. If symptoms persist unabated, the use of complement inhibitors such as Eculizumab is advised [38]. These steps are designed to ensure the safety and efficacy of treatment, managing the risk of TMA through a stepwise intervention approach.

## 5 METHODS

### Clinical database analysis

#### Source of clinical database

This observational, retrospective pharmacovigilance study analyzes AEs associated with TMA in patients treated with specific VEGF inhibitors (Bevacizumab, Ranibizumab, Brolucizumab, Aflibercept, Conbercept, Pegaptanib) and VEGFR inhibitors (Ramucirumab, Nintedanib, Apatinib, Axitinib, Sunitinib, Sorafenib, Regorafenib, Vandetanib, Cabozantinib, Pazopanib, Lenvatinib, Anlotinib, Fruquintinib, Tivozanib, Cediranib, Brivanib) [43]. Data were sourced from two primary pharmacovigilance databases: the FAERS and the WHO's global drug monitoring database, Vigibase. These databases provide extensive post‐market safety data, capturing a wide range of information, including demographics, drug usage, AEs, and outcomes from 2013 to 2023 for FAERS, and from 1968 to 2023 for Vigibase [44]. Both systems are instrumental in collecting individual case safety reports from healthcare professionals and consumers across over 130 countries. This global coverage ensures a comprehensive analysis of spontaneous report data. Adverse events are meticulously classified using the Medical Dictionary for Regulatory Activities system, which includes Preferred Terms (PTs) [45], Higher‐Level Terms, and aggregates into the System Organ Class, providing a structured framework for detailed pharmacovigilance investigations.

#### Clinical database processing procedure

Initially, we removed duplicate entries from the FAERS database, filtering for exact matches in gender, age, country, event date, adverse events, medication, and indications to ensure data accuracy [46]. We focused on oncology‐related cases, analyzing 1,566,519 patients, including 131,586 treated with VEGF or VEGFR inhibitors. In Vigibase, we used the “SUSPECTEDDUPLICATES” data set to eliminate duplicates, focusing on 1,552,132 reports linked to cancer. We standardized drug names using medicinal product codes and extracted data for 151,742 patients receiving VEGF or VEGFR inhibitors [47].

#### Clinical database signal mining

In this study, we compiled the brand names of VEGF and VEGFR inhibitors (Table S1) and collected six AEs associated with TMA for in‐depth analysis using the disproportionality analysis method ROR [48]. By using ROR and its 95% confidence interval (CI), we can quantify the statistical significance and association strength of these adverse event signals.

If the number of reports for a particular adverse event is not less than three, and the lower bound of its 95% CI for ROR exceeds one, we consider these adverse events to be highly associated with the use of VEGF inhibitors.

#### Local patient data analysis

We conducted a retrospective analysis of 1698 patients treated with VEGF or VEGFR inhibitors at Southern Medical University's Zhujiang Hospital from 2012 to 2023 (Table S2). Inclusion criteria were: (1) confirmed diagnosis of malignant tumors; (2) received VEGF or VEGFR inhibitor therapy between 2012 and 2023; (3) had complete biochemical marker data recorded both before and after treatment, with consistent testing times. Exclusion criteria included: (1) concurrent use of other known TMA‐inducing medications; (2) history of TMA; (3) incomplete biochemical marker data or interruptions in treatment during the study period. We focused on key biochemical indicators—platelet count, creatinine, indirect bilirubin, RBC, and MCV—to evaluate the risk of TMA and TTP [49]. Measurements were taken at consistent time points before and after treatment, with baseline values established for accurate assessment of treatment effects. We used Z‐score transformations to standardize the data and minimize outliers, summing the log_2_‐transformed Z‐scores of the indicators to calculate an overall risk score [50]. Patients with platelet counts below 30 × 10^9/L and creatinine levels below 199 μmol/L were classified as high risk for TTP [51], based on the French scoring criteria. The study protocol was approved by the Ethics Committee of Southern Medical University's Zhujiang Hospital [52].

#### Calculation of enrichment scores for biological pathways in pan‐cancer TCGA

To investigate the molecular mechanisms of TMA linked to VEGF and VEGFR inhibitors, we accessed transcriptome data from 35 cancer types in the UCSC Xena database, part of the TCGA project. Expression data were converted from FPKM to TPM format, and we conducted ssGSEA using the GSVA package [53]. Enrichment scores for pathways from the MSigDB database—covering Gene Ontology, Kyoto Encyclopedia of Genes and Genomes, and Reactome—were calculated for each cancer sample. This analysis helped identify potential biological mechanisms by correlating the Reporting ROR of TMA‐related adverse events with pathway activities across various cancers.

### Animal experiment analysis

#### Experimental grouping

In a study approved by the Animal Experimentation Center of Zhujiang Hospital of Southern Medical University (IACUC‐SAHCQMU‐2023‐0044), we used 48 male C57BL/6J mice, aged 6–8 weeks and weighing 25 g, provided by Jiangsu Huachuang Xinnuo Pharmaceutical Technology Co., Ltd. The mice had free access to food and water and were divided into two principal groups: the Chronic Nephropathy (CN) and the Acute Nephropathy (AN) models, each with 24 mice. These groups were subdivided into four: PBS control, Bevacizumab, DMSO control, and Semaxanib, with six mice each. In the CN model, Bevacizumab and Semaxanib were given at 5 and 10 mg/kg, respectively, twice weekly for 4 weeks. In the AN model, the doses were doubled to 10 and 20 mg/kg, respectively, administered similarly over 2 weeks.

#### Kidney tissue processing and analysis

At the conclusion of the experiment, all mice were euthanized, and their kidneys were rapidly harvested for further analysis [54]. The kidneys were bisected; one half was designated for histological analysis, fixed in 10% neutral buffered formalin, dehydrated, embedded in paraffin, and sectioned into 5 µm thick slices for H&E staining. The other half of the kidney tissue remained unfixed and was immediately frozen in liquid nitrogen to preserve RNA integrity. After thawing, total RNA was extracted. Only RNA that met quality and concentration standards was used for cDNA synthesis and subsequent high‐throughput sequencing to analyze gene expression changes postdrug treatment [55].

### Statistical analysis

In this study, we analyzed potential risk factors such as age, gender, and type of medication using logistic regression models [56]. For variables that followed a normal distribution, parametric tests such as the independent samples *t*‐test were employed. For variables that did not meet the normality assumption, non‐parametric tests such as the Mann–Whitney *U* test were utilized. We plotted cumulative distribution functions to examine the temporal distribution of TMA‐related AEs caused by VEGF and VEGFR inhibitors, with the Mann–Whitney *U* test determining the statistical differences in onset timing. Biochemical markers from patients at Southern Medical University's Zhujiang Hospital were assessed to compare changes before and after medication and evaluate TMA/TTP risk using Mann–Whitney *U*, *G* tests, and Fisher's tests. Differential gene expression analysis was conducted to identify differences between experimental and control groups in mouse sequencing data, followed by GSEA to investigate relevant biological pathways. Associations between pathway activations and drug‐induced AEs were explored using single‐sample GSEA and Spearman's rank correlation coefficients with TCGA pan‐cancer pathway data. All statistical analyses, including a two‐sided *p*‐value threshold of less than 0.05 for significance, were performed using R software (version 4.3.1). Data processing and figure visualizations were also conducted in R.

## AUTHOR CONTRIBUTIONS

**Aimin Jiang**: Investigation; methodology; formal analysis; project administration; conceptualization. **Zhanzhi Li**: Writing—original draft; visualization; methodology; software. **Ying Liu**: Formal analysis; project administration. **Junyi Shen**: Validation; software; data curation; supervision; investigation. **Quan Cheng**: Writing—review and editing; investigation; validation; resources. **Anqi Lin**: Data curation; project administration; supervision; writing—review and editing; conceptualization; visualization. **Peng Luo**: Project administration; data curation; supervision; conceptualization; resources. **Linhui Wang**: Supervision; project administration; writing—review and editing; conceptualization; funding acquisition.

## CONFLICT OF INTEREST STATEMENT

The authors declare that the research was conducted in the absence of any commercial or financial relationships that could be construed as a potential conflict of interest.

## 1 ETHICS STATEMENT

The animal experiment protocol of this study was in accordance with the animal care and use committee of the Zhujiang Hospital of Southern Medical University and the guiding principles of animal experimentation of the institute (LAEC‐2024‐022). The clinical study protocol was approved by the Ethics Committee of Southern Medical University's Zhujiang Hospital (2024‐KY‐129‐01).

## Supporting information


**Figure S1.** TTO analysis of gender.
**Figure S2.** Complications of TMA.

**Table S1.** Drug name.
**Table S2.** Patient examination indicator data.
**Table S3.** Demographics of TMA Patients Treated with VEGF or VEGFR Inhibitors in FAERS and Vigibase.
**Table S4.** ROR data.

## Data Availability

The data that supports the findings of this study are available in the supplementary material of this article. The clinical data from the FAERS and Vigibase databases are publicly available and can be accessed through their respective official websites. The local patient data used in this study are available in Table S3. The data and scripts used are saved in GitHub (https://github.com/lizhanzhi/VEGFi_TMA). Supplementary materials (figures, tables, graphical abstract, slides, videos, Chinese translated version, and update materials) may be found in the online DOI or iMetaOmics (http://www.imeta.science/imetaomics/).
