# Supplementary material for: Linking preclinical models to clinical realities: VEGF/VEGFR inhibitors and thrombotic microangiopathy in cancer therapy
Source: IMetaOmics. 2025 Mar 28;2(2):e70014. doi: 10.1002/imo2.70014 (PMC12806200; doi:10.1002/imo2.70014)
Supplement: Supplementary file 1 — Figure S1. TTO analysis of gender. Figure S2. Complications of TMA. [file IMO2-2-e70014-s001.DOCX]

**Supporting information to:**

**Linking preclinical models to clinical realities: VEGF/VEGFR inhibitors and thrombotic microangiopathy in cancer therapy**

Aimin Jiang^1#^, Zhanzhi Li^2#^, Ying Liu^1#^, Junyi Shen^3#^, Quan Cheng^4,5*^, Anqi Lin^3*^, Peng Luo^3*^, Linhui Wang^1*^

^1^Department of Urology, Changhai Hospital, Naval Medical University (Second Military Medical University), Shanghai 200433, China

^2^School of Clinical Medicine, Hangzhou Medical College, Hangzhou 310053, China

^3^Department of Oncology, Zhujiang Hospital, Southern Medical University, Guangzhou 510282, China

^4^Department of Neurosurgery, Xiangya Hospital, Central South University, Changsha 410008, Hunan, China

^5^National Clinical Research Center for Geriatric Disorders, Xiangya Hospital, Central South University, Changsha 410008, Hunan, China

**^#^**These authors contributed equally: Aimin Jiang, Zhanzhi Li, Ying Liu, Junyi Shen

^*^Correspondence: [chengquan@csu.edu.cn](mailto:chengquan@csu.edu.cn) (Quan Cheng), [smulinanqi0206@i.smu.edu.cn](mailto:smulinanqi0206@i.smu.edu.cn) (Anqi Lin), [luopeng@smu.edu.cn](mailto:luopeng@smu.edu.cn) (Peng Luo) & [wanglinhui@smmu.edu.cn](mailto:wanglinhui@smmu.edu.cn) (Linhui Wang)

**Supplementary figures:**


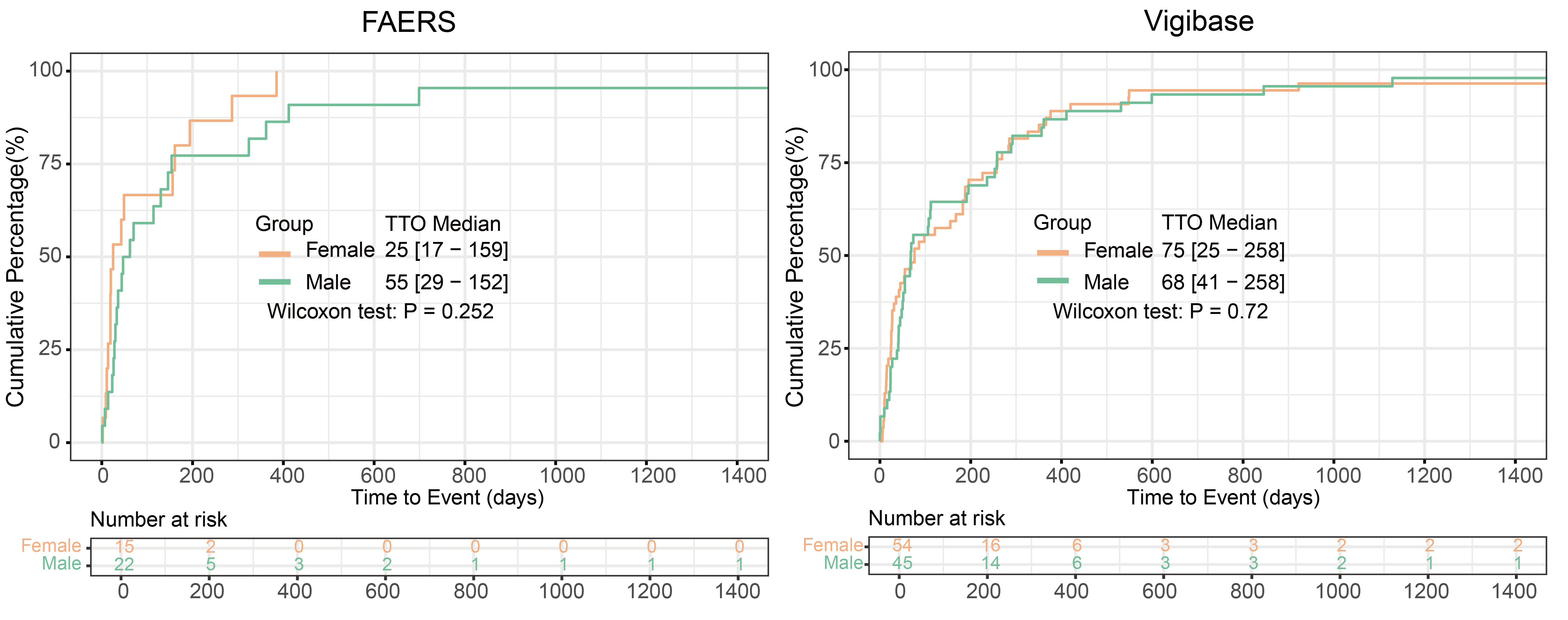


**Figure S1.** Cumulative percentage plots show differences in the distribution of time to thrombotic microangiopathy (TMA) in patients of different sexes treated with vascular endothelial growth factor (VEGF) and vascular endothelial growth factor receptor (VEGFR) inhibitors in the FDA Adverse Event Reporting System (FAERS) and the WHO Global Database for Adverse Drug Reactions (Vigibase).

**Figure S2.** Dendrogram of concurrent adverse reactions related to TMA adverse reactions.
